# Statistics of correlated percolation in a bacterial community

**DOI:** 10.1371/journal.pcbi.1007508

**Published:** 2019-12-02

**Authors:** Xiaoling Zhai, Joseph W. Larkin, Kaito Kikuchi, Samuel E. Redford, Ushasi Roy, Gürol M. Süel, Andrew Mugler

**Affiliations:** 1 Department of Physics and Astronomy, Purdue University, West Lafayette, Indiana, United States of America; 2 Division of Biological Sciences, University of California San Diego, La Jolla, California, United States of America; 3 San Diego Center for Systems Biology, University of California San Diego, La Jolla, California, United States of America; Princeton University, UNITED STATES

## Abstract

Signal propagation over long distances is a ubiquitous feature of multicellular communities, but cell-to-cell variability can cause propagation to be highly heterogeneous. Simple models of signal propagation in heterogenous media, such as percolation theory, can potentially provide a quantitative understanding of these processes, but it is unclear whether these simple models properly capture the complexities of multicellular systems. We recently discovered that in biofilms of the bacterium *Bacillus subtilis*, the propagation of an electrical signal is statistically consistent with percolation theory, and yet it is reasonable to suspect that key features of this system go beyond the simple assumptions of basic percolation theory. Indeed, we find here that the probability for a cell to signal is not independent from other cells as assumed in percolation theory, but instead is correlated with its nearby neighbors. We develop a mechanistic model, in which correlated signaling emerges from cell division, phenotypic inheritance, and cell displacement, that reproduces the experimentally observed correlations. We find that the correlations do not significantly affect the spatial statistics, which we rationalize using a renormalization argument. Moreover, the fraction of signaling cells is not constant in space, as assumed in percolation theory, but instead varies within and across biofilms. We find that this feature lowers the fraction of signaling cells at which one observes the characteristic power-law statistics of cluster sizes, consistent with our experimental results. We validate the model using a mutant biofilm whose signaling probability decays along the propagation direction. Our results reveal key statistical features of a correlated signaling process in a multicellular community. More broadly, our results identify extensions to percolation theory that do or do not alter its predictions and may be more appropriate for biological systems.

## Author summary

Many multicellular systems send signals over long distances by relaying information over connected cell-to-cell paths. In physics, the statistics of connected path formation are described by percolation theory. We previously discovered that the statistics of electrical signal propagation in communities of the bacterium *Bacillus subtilis* are consistent with the predictions of percolation theory. However, we find experimentally that key features of this system go beyond the simple assumptions of basic percolation theory, which include site-to-site independence and spatial uniformity of the signaling probability. Why are the predictions of percolation theory still upheld? Using a computational model, we find that the cell-to-cell dependence does not change the predictions due to the universal nature of percolation theory near its critical point, and the spatial variability of the signaling probability actually expands the parameter range over which the predictions hold. We validate our findings using a mutant bacterial strain. Our work explores the robustness of percolation theory to its underlying assumptions, and the resulting consequences for long-range bacterial signaling.

## Introduction

Long-range signal transmission is central to the function of many multicellular communities. However, cell-to-cell variability within these communities [1, 2] can cause some cells not to participate in signaling, which may degrade or attenuate the signal [3–5]. In physics, signal transmission in the presence of non-propagating agents is the domain of percolation theory [6]. As a result, many investigators have turned to percolation theory to describe signal transmission in multicellular systems. In bacterial communities, percolation theory has been used to predict the scaling laws that result from signal disruption during quorum sensing [7]. In neuroscience, percolation theory has been used to describe (i) the transition from a fully connected to a disconnected electrical network in rat hippocampus cultures [8, 9], (ii) the spatiotemporal structure of viral propagation within astrocyte monolayers [10], and (iii) the transition from conscious to unconscious brain activities during general anesthesia [11]. In pancreatic islets, percolation theory has been used to understand the dependence of calcium wave propagation on the coupling strength of gap junctions between the islet cells [12]. In colonies of *Spirostomum* (an aquatic worm-like cell), percolation theory was recently shown to describe how the propagation of a hydrodynamic cell-to-cell trigger-wave depends on the colony density [13].

We recently demonstrated that the transmission of an electrical signal from the interior to the periphery of a biofilm of *Bacillus subtilis* bacteria is consistent with the predictions of percolation theory [5]. In this system, starvation of the interior cells causes release of intracellular potassium, which leads to depolarization and potassium release in neighboring cells, resulting in a cell-to-cell relay wave that propagates to the biofilm periphery [14–16]. The signal temporarily prevents peripheral cells from taking up nutrients and thus allows nutrients to diffuse to the interior cells, preserving biofilm viability and increasing its overall fitness [14]. However, it turns out that not all cells participate in the potassium release: we discovered that the fraction of participating cells is near the percolation threshold, and that clusters of participating cells have a size distribution that follows a power law with an exponent predicted by percolation theory [5]. Operating near the percolation threshold allows the biofilm to maintain successful signal transmission while minimizing the number of cells that undergo the costly potassium release [5].

Despite the success of percolation theory as a description of signal transmission within this system, it is reasonable to suspect that several key assumptions of percolation theory may require scrutiny in this and many similar multicellular systems [5]. First, percolation theory assumes that the probability for each cell to participate in signal transmission is independent of other cells. However, in reality it may be that the participation probability of a cell is correlated with that of its neighbors. For example, if the molecular mechanism governing participation is heritable, then one expects the participation of a given cell to be correlated with other cells in its lineage, which are most likely to be nearby in the densely packed biofilm. Second, percolation theory assumes that the participation probability does not vary from one biofilm to another, or from location to location within a biofilm. However, in reality we know that there is variability across biofilms, and particular mutant strains have spatial variability in the participation fraction [5]. These considerations raise the question of when and how percolation theory remains a predictive description of signal transmission in biological systems. Conversely, they suggest a strategy by which deviations from percolation theory would give important insights about the ways in which a biological system differs from the model assumptions [17]. They also raise the broader question of which predictions of a model from statistical physics are dependent on the model details, and which predictions are universal.

Here we use a combination of simulations and experiments to investigate the statistical properties of signal percolation in a bacterial biofilm. We find that signal correlations exist between cells, due to a combination of phenotypic inheritance and spatial proximity of a cell to its progeny. We find that while these correlations lower the percolation threshold, they are not sufficiently long-range to affect the cluster size statistics. Instead, we find that variability in the signaling fraction within and across biofilms affects the statistics by widening the range of fractions at which one observes the power-law distribution of cluster sizes. We validate our findings using a mutant biofilm whose participation fraction decays as a function of propagation distance. Our results demonstrate that certain community-level signaling properties are robust to cell-level features whereas others are not, and we discuss the implications for biofilm function.

## Results

**Fig 1:**
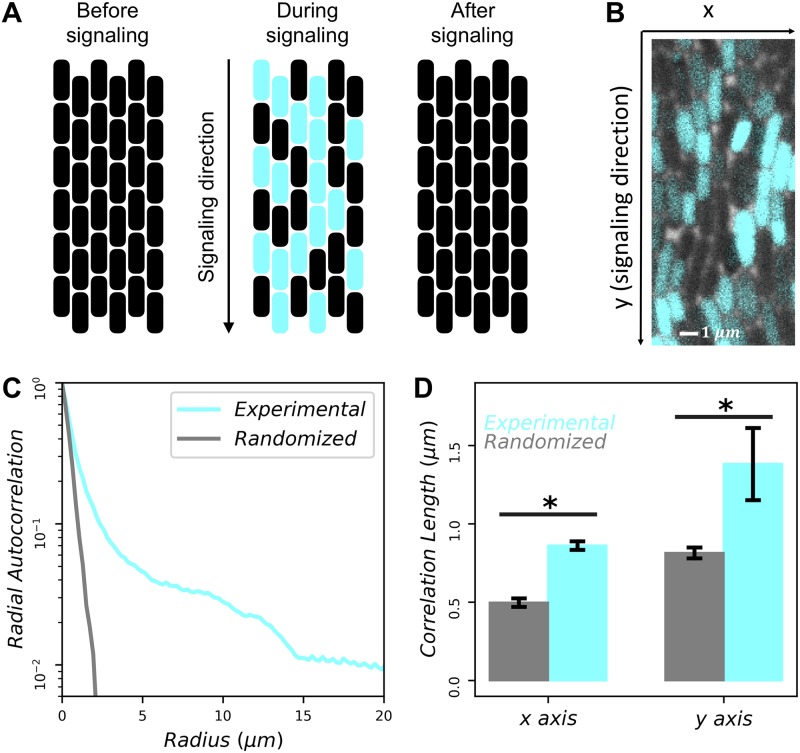
Signaling probability of each cell is correlated with neighboring cells. (A) Cartoon illustrating electrical signaling wave transmitted across biofilm. Cyan represents cells that participate in signaling. (B) Zoomed-in snapshot of cells in biofilm during peak of signal transmission (actual experimental window is approximately 35 cells tall by 230 cells wide). Cyan indicates fluorescence intensity of ThT dye, proportional to membrane potential. (C) Correlation function is longer-range than that from randomized data (*N* = 3 biofilms). (D) Correlations are significantly longer than random both perpendicular (*x*) and parallel (*y*) to the signaling direction (*p* < 0.001 and *p* = 0.007 assuming Gaussian errors, respectively).

We first review the key features of electrical signaling in the biofilm [5, 14–16], and those of percolation theory, as these features will motivate our present results. The electrical signal is transmitted by cells across the biofilm in a wave-like manner (Fig 1A). We measure the membrane potential of cells during the peak of signal transmission using a fluorescent dye (cyan in Fig 1B; see Materials and methods). We previously observed a bimodal distribution of dye intensity across cells [5], which provides a threshold above or below which we define cells as “on” (participating in the signal) or “off” (not participating in the signal), respectively. This observation motivates our use of percolation theory, as percolation theory describes the connectivity and spatial statistics of systems on a lattice in which each cell has a probability *ϕ* to be on.

We alert the reader that in typical applications of percolation theory, one can measure both the input (the ability of each component to signal or not) and the output (whether or not each component actually participates in the signal as it propagates). Here, because we do not know the molecular mechanism that confers the ability to signal, we can only measure the output. Nonetheless, we observe in [5] that (i) isolated clusters participate in signaling, and (ii) the percolation threshold remains predictive of whether the signal propagates across the biofilm. Therefore, as in [5], we conclude that the signaling mechanism is sufficiently short-range that percolation is a relevant criterion for propagation, but sufficiently long-range that the output can be treated as a reasonably good proxy for the input.

Our experiments focus on a 2D cell monolayer at the edge of the biofilm (see Materials and methods). We previously found that cells are most likely to have six neighbors [5]. For an infinite 2D, six-neighbor lattice, percolation theory predicts that (i) a connected path of on-cells emerges above the critical value *ϕ*_*c*_ = 1/2, and that (ii) at *ϕ*_*c*_, the distribution of on-cell cluster sizes *P*(*n*) becomes a power law [6].

In the experiments, we image a finite window of approximately 35 by 230 cells (see Materials and methods). Finite-size effects can change the value of *ϕ*_*c*_ at which connectivity sets in, which we call $\phi_{c}^{\text{conn}}$ [6]. Indeed, simulations predict that $\phi_{c}^{\text{conn}}=0.45$ in this finite geometry [5]. Finite-size effects should not change the value of *ϕ*_*c*_ at which *P*(*n*) becomes a power law, which we call $\phi_{c}^{\text{pow}}=1/2$, so long as $\sqrt{n}$ is sufficiently below the smaller lattice dimension. However, at larger *n* values the distribution will deviate from a power law, even at $\phi_{c}^{\text{pow}}$, due to finite-size effects.

We previously observed that the fraction of on-cells in the experiments is *ϕ* = 0.43 ± 0.02 (mean ± standard error), and that the distribution *P*(*n*) of on-cell cluster sizes is a power law over three decades [5]. The fact that $\phi \approx \phi_{c}^{\text{conn}}$ suggests that the system sits at the connectivity threshold. However, the fact that $\phi <\phi_{c}^{\text{pow}}$ raises the question of why a power law is observed, particularly one with no apparent finite-size effects at large *n*. To address this question, as well as the broader question of what features of percolating systems are expected to be robust to the underlying assumptions about the components, we now investigate the effects of signal correlations and of variability in the signaling fraction.

### Participation in signaling is spatially correlated

Percolation theory assumes that a fraction *ϕ* of on-cells are situated randomly in space. However, in the biofilm one might expect that on-cells are spatially co-located, for example if participating in the signal is a heritable phenotype. To determine whether there are spatial correlations in on-cells, we measure the radial autocorrelation function

$$\begin{array}{c} C\left(r\right)={\left\langle s_{i}s_{j}\right\rangle }_{r}-\phi^{2}, \end{array}$$ (1)

where *s* = 1 for on-cells, *s* = 0 for off-cells, and the average is taken over all pixels *i* and *j* whose separation is *r* (see Materials and methods). We find that *C*(*r*) is a decreasing function of *r*, as expected (Fig 1C, cyan curve). We then compare *C*(*r*) to the autocorrelation function computed with the locations of on-cells randomized. Specifically, we retain the locations of all cells and the number of on-cells, but we randomize which cells are on (as would be the case in percolation theory). We see in Fig 1C that *C*(*r*) falls off more steeply in this case (gray curve). These results suggest that on-cells are more spatially correlated than expected from random placement.

We next investigate the strength of correlation perpendicular (*x*) and parallel (*y*) to the direction of signal transmission (Fig 1B). We define the correlation lengths as *ξ*_*x*_ = ∫*dx* *C*(*x*) and *ξ*_*y*_ = ∫*dy* *C*(*y*), where *C*(*x*) and *C*(*y*) are defined as in Eq 1 but restricted to separations perpendicular (*x*) or parallel (*y*) to the signaling direction, and the integrals run from zero to the maximal separation values. Even in the randomized data, we see that the correlation length is larger in the *y* direction than in the *x* direction (compare the gray bars in Fig 1D) because cells are longer than they are wide, and the long axis of each cell is generally oriented in the signaling direction (Fig 1B). In the actual (non-randomized) data, the correlation lengths are 70% larger than random in both the *x* and *y* directions, and both differences are significant (*p* < 0.01; Fig 1D). These results suggest that on-cells are significantly correlated both parallel and perpendicular to the signaling direction.

To quantify the correlation at the single-cell level, we consider the conditional probabilities *p*(on|on) and *p*(off|off), where *p*(on|on) is the probability that a cell is on given that the cell above it is also on, and similarly for *p*(off|off). We then calculate the order parameter

$$\begin{array}{c} \rho =p(\text{on}|\text{on})-p(\text{on}|\text{off}), \end{array}$$ (2)

where *p*(on|off) = 1 − *p*(off|off). With no correlation, we have *p*(on|on) = *p*(on|off) = *ϕ*, and therefore *ρ* = 0. With perfect correlation, we have *p*(on|on) = 1 and *p*(on|off) = 0, and therefore *ρ* = 1. Thus, *ρ* quantifies the cell-to-cell correlation in the signaling direction on a scale from zero to one.

**Fig 2:**
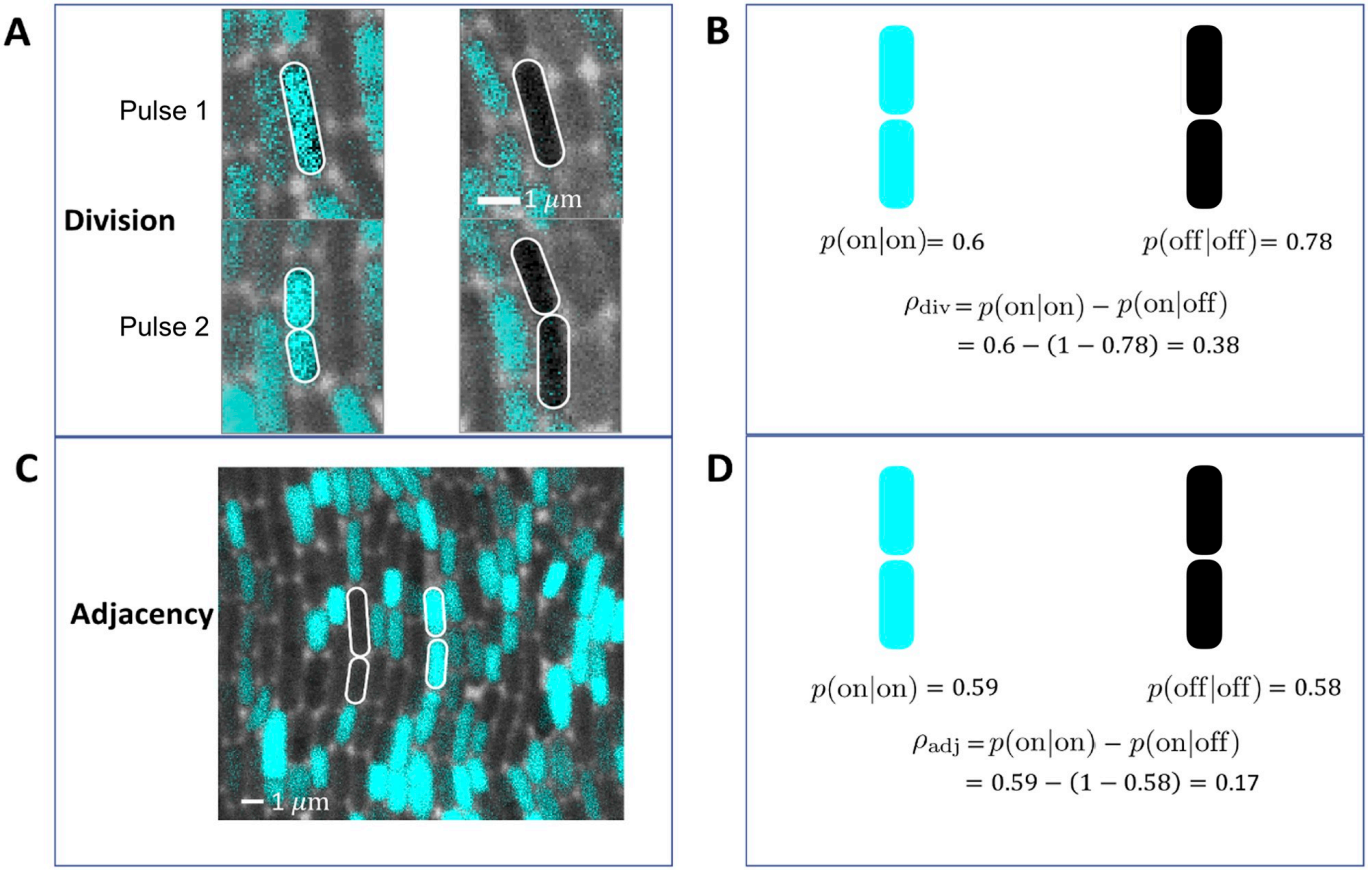
Order parameter *ρ* quantifies degree of spatial correlations. (A, B) Lineage-tracing experiments yield *ρ*_div_ = 0.38 (*N* = 49 division events). (C, D) Spatial analysis of the biofilm images yield *ρ*_adj_ = 0.17 (*N* = 51 cell pairs).

We estimate the conditional probabilities, and thus *ρ*, in two ways (Fig 2). First, because cell division is usually parallel to the signaling direction, we track division events that occur in between signal pulses (Fig 2A; see Materials and methods). We then count the number of times that the top cell has the same or different signaling state as the bottom cell. From this method we obtain *ρ*_div_ = 0.38 (Fig 2B). Second, we estimate the conditional probabilities directly from pairs of cells that are adjacent to each other in the signaling direction during signaling (Fig 2C; see Materials and methods). From this method we obtain *ρ*_adj_ = 0.17 (Fig 2D). These results confirm at the single-cell level that spatial correlations exist in the signaling direction (*ρ*_adj_ > 0) but suggest that these correlations are less strong than those produced directly by division (*ρ*_adj_ < *ρ*_div_).

### Mechanistic model of correlated signaling

To understand the experimental results above, we propose a mechanistic model of spatially correlated cell signaling. We hypothesize that the signaling state is heritable during cell division with a certain probability, and that cell displacement can occur at the leading edge as the biofilm grows. The assumption that cells possess a signaling state variable is supported by the observation that a cell generally does not switch its on/off signaling behavior between successive pulses in the experiments [5].

**Fig 3:**
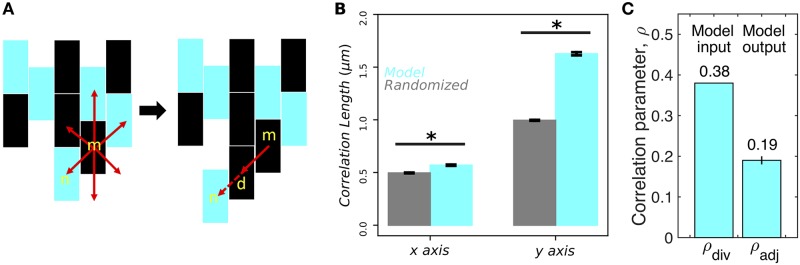
Mechanistic model of correlated signaling captures experimental features. (A) Mother cell (m) produces daughter cell (d) with correlated signaling state at any neighboring site at which a maximum of one neighbor cell (n) is displaced. Cyan indicates that cell has the ability to signal. (B) Correlations are significantly longer than random both perpendicular (*x*) and parallel (*y*) to the signaling direction (*N* = 10^4^ lattices; *p* < 0.001 for both assuming Gaussian errors). Compare to experiments in Fig 1D. (C) Stochasticity in division times, neighbor selection, and cell displacement reduces correlation parameter from *ρ*_div_ = 0.38 to *ρ*_adj_ = 0.19 ± 0.01, close to experimentally measured *ρ*_adj_ = 0.17 (*N* = 10^4^ lattices).

Specifically, as shown in Fig 3A, we generate a 2D, six-neighbor lattice of rectangular cells with aspect ratio 2 (the approximate experimental value) in the following way. Each cell divides after a time *τ* drawn from a Gaussian distribution with mean $\bar{\tau }$ and standard deviation *δτ*. The “mother” cell (m) retains its location and signaling state, while the “daughter” cell (d) occupies one of the eligible neighboring locations with equal probability. Eligibility requires that the neighboring location either be empty or be occupied by a neighboring cell (n) that, when displaced by the division along the same direction, would occupy an empty location (Fig 3A). Because the biofilm is growing downward, the eligible locations will most often be the location directly below and, with lower probability, the locations below-and-to-the-right and below-and-to-the-left. The signaling state of the daughter, given that of the mother, is determined from the division parameter *ρ*_div_ and the fraction of on-cells *ϕ* according to

$$\begin{array}{c} p(\text{on}|\text{on})=\phi +\rho_{\text{div}}-\phi \rho_{\text{div}}, \end{array}$$ (3)

$$\begin{array}{c} p(\text{on}|\text{off})=\phi -\phi \rho_{\text{div}}, \end{array}$$ (4)

which follow from Eq 2 and the requirement that the fraction of on-cells remains *ϕ* throughout the process (see Materials and methods). We produce a 100 by 230 lattice of cells by initializing the top row randomly and generating the next 99 rows according to the above mechanism. Then we remove the top 55 and bottom 10 rows, leaving a 35 by 230 cell window as in the experiments. This procedure allows the mechanism to achieve statistical steady state and focuses on the biofilm edge as in the experiments.

We find that the spatial statistics are not sensitive to the value of $\delta \tau /\bar{\tau }$, so long as it is greater than zero, and therefore we average our results over the range $0<\delta \tau /\bar{\tau }<1$ (rejecting samples with *τ* ≤ 0 for large *δτ*). We also find that allowing neighbor cell displacement is necessary to generate correlations in the *x* direction, but that allowing two or more levels of displacement does not qualitatively change the results. Thus, the only parameters in the model are *ϕ* and *ρ*_div_, which we set from the experiments as *ϕ* = 0.43 [5] and *ρ*_div_ = 0.38 (Fig 2B).

This model, with no free parameters, makes three predictions. Specifically, the model predicts that (i) the correlation length in the *x* direction is significantly different from random (Fig 3B), (ii) the correlation length in the *y* direction is significantly different from random (Fig 3B), and (iii) the spatial correlation parameter measured from adjacent cells in the *y* direction after the biofilm is generated is *ρ*_adj_ = 0.19 ± 0.01 (Fig 3C). The model output *ρ*_div_ is reduced from the model input *ρ*_div_ = 0.38 due to the stochasticity in division times, neighbor selection, and cell displacement. Predictions (i) and (ii) are consistent with the experiments, as both the *x* and *y* correlation lengths were found to be significantly different than random (Fig 1D). Prediction (iii) is also consistent with the experiments, as *ρ*_adj_ was measured to be 0.17 (Fig 2D), which is very close to 0.19 ± 0.01. We have also checked that these predictions remain unchanged when accounting for the fact that on-cells grow more slowly than off-cells [5] (see Materials and methods). The fact that all three predictions are validated by the experiments gives us confidence that the model captures the basic underlying mechanism, especially because it has no free parameters.

### Impact of correlations on spatial statistics

**Fig 4:**
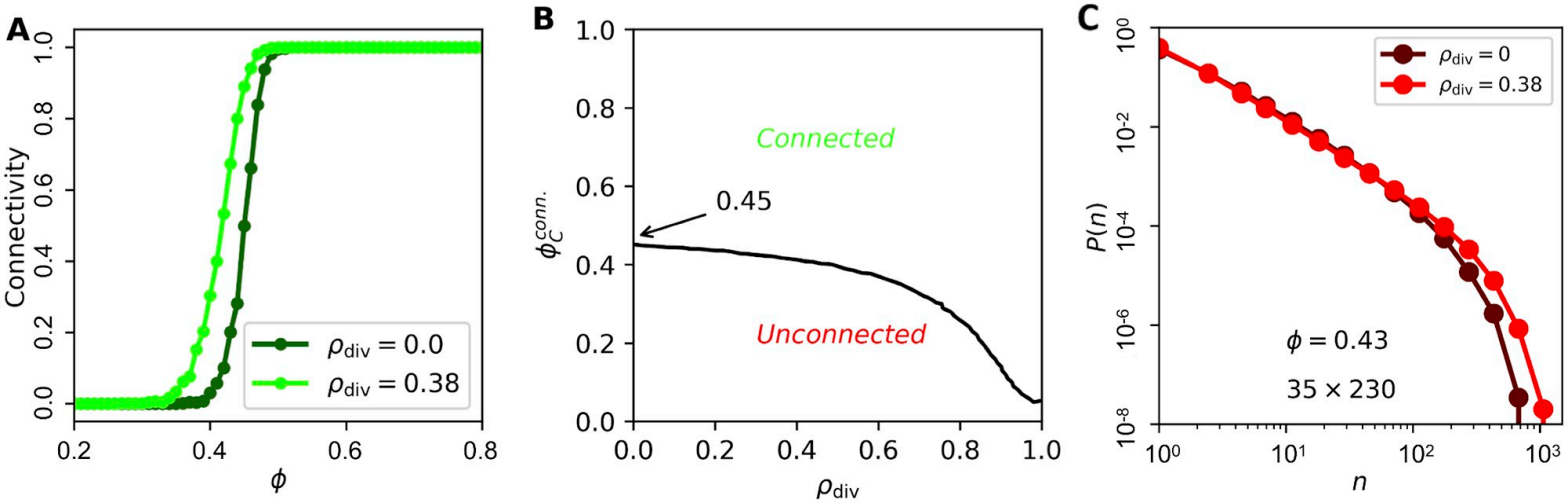
Spatial correlations increase connectivity but have little effect on cluster size distribution. (A) Connectivity, defined as probability that a connected path of on-cells exists, occurs at lower on-cell fraction *ϕ* as correlation parameter *ρ*_div_ increases (*N* = 10^3^ lattices). (B) Connectivity threshold $\phi_{c}^{\text{conn}}$, defined as *ϕ* value for which connectivity is 50%, decreases with *ρ*_div_ (*N* = 10^3^ lattices). (C) Spatial correlations (*ρ*_div_ = 0.38) have little effect on distribution, in particular not removing exponential rolloff at large *n* (*N* = 10^3^ lattices).

We now use our mechanistic model to investigate the impact of the spatial correlations on the statistical properties of the biofilm. First we focus on the connectivity: the probability, over an ensemble of simulated biofilms, that a connected path of on-cells exists from the top to the bottom of the lattice. The connectivity is expected to show a sharp transition from 0 to 1 at a critical fraction of on-cells $\phi_{c}^{\text{conn}}$. For an infinite lattice (in 2D with six neighbors), $\phi_{c}^{\text{conn}}=1/2$ [6]. Finite-size effects reduce the sharpness, but $\phi_{c}^{\text{conn}}$ can still be defined as the value of *ϕ* for which the connectivity is 50%. For a finite lattice of the approximate size of the experimental window (35 cells tall by 230 cells wide), without correlations, we previously found $\phi_{c}^{\text{conn}}=0.45$ [5] (Fig 4A, dark green curve). With correlations, using our mechanistic model with *ρ*_div_ = 0.38, we find $\phi_{c}^{\text{conn}}=0.4$ (Fig 4A, light green curve). More generally, the connectivity threshold is shown as a function of *ρ*_div_ in Fig 4B, and we see that as $\rho_{\text{div}}\to 1$, $\phi_{c}^{\text{conn}}$ becomes close to zero, even with the stochasticity inherent in the model. Thus, spatial correlations reduce the connectivity threshold. This makes sense, as correlations increase the probability of connected on-cells, particularly in the signaling direction, and this lowers the fraction of on-cells needed to created a connected path.

Second, we investigate the impact of correlations on the distribution of on-cell cluster sizes *P*(*n*). The distribution is expected to become a power law at a critical fraction of on-cells $\phi_{c}^{\text{pow}}=1/2$ [6]. The experimental fraction of on-cells is *ϕ* = 0.43 ± 0.02 [5], which is lower than $\phi_{c}^{\text{pow}}$. In simulations without correlations, at *ϕ* = 0.43, we find that *P*(*n*) acquires a rolloff (when viewed on a log-log scale) at large *n* (Fig 4C, dark red curve). The rolloff indicates that the distribution is becoming more exponential, as expected for $\phi <\phi_{c}^{\text{pow}}$. However, in experiments, we find that *P*(*n*) maintains the power law dependence, with no rolloff, for three decades, i.e. out to *n* = 10^3^ [5]. Because we have seen that spatial correlations preserve connectivity at lower *ϕ* (Fig 4A and 4B), we hypothesize that correlations may also preserve the power law dependence of *P*(*n*) at lower *ϕ*, and thus explain the experimental observation. Surprisingly, using our mechanistic model, we find that the spatial correlations actually have little impact on *P*(*n*) (Fig 4C, light red curve): the rolloff is slightly shifted to larger *n*, but it is certainly still present over the three-decade range.

**Fig 5:**
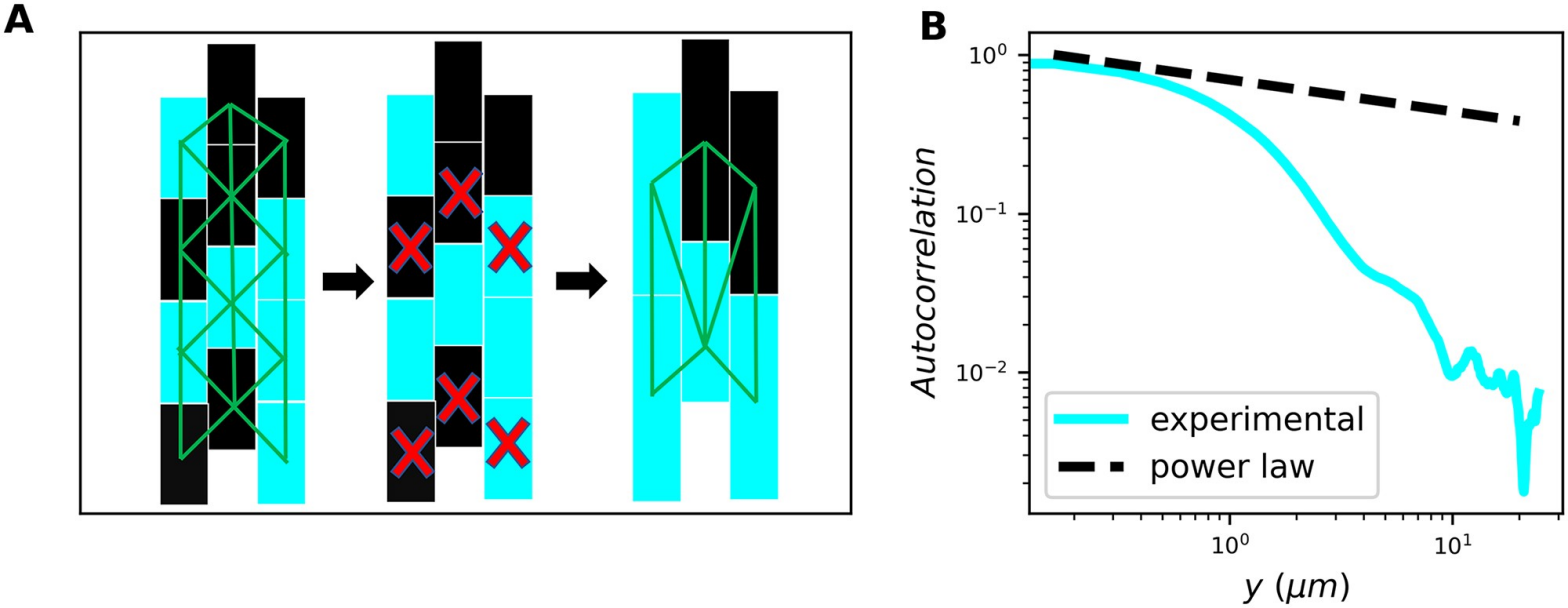
Short-range correlations do not affect critical properties. (A) Illustration of the renormalization argument: upon site decimation, lattice remains triangular, *ϕ* remains constant, and *ρ* vanishes. (B) Correlation function in experiments is short-range, i.e. sub-power-law (*N* = 3 biofilms).

Why do correlations not change the distribution of cluster sizes? Renormalization-group arguments from statistical physics imply that correlations do not change the critical properties of percolation theory if the correlations are sufficiently short-range [18]. The intuitive reason can be seen from a site-decimation procedure [6], as illustrated in Fig 5A. We imagine decimating every other cell in each column (red X’s), with each remaining cell expanding to fill the space below it. Fig 5A illustrates that the resulting lattice remains triangular (green lines). Furthermore, because the probability of any cell to be on is *ϕ*, the fraction of on-cells remains *ϕ* after decimation. Finally, the new conditional probabilities after one round of decimation are

$$\begin{array}{c} p_{1}\left(\text{on}|\text{on}\right)=p(\text{on}|\text{on})p(\text{on}|\text{on})+p(\text{on}|\text{off})p(\text{off}|\text{on}), \end{array}$$ (5)

$$\begin{array}{c} p_{1}\left(\text{on}|\text{off}\right)=p(\text{on}|\text{on})p(\text{on}|\text{off})+p(\text{on}|\text{off})p(\text{off}|\text{off}), \end{array}$$ (6)

which follow from the rules of probability and the assumption that the signaling state is spatially Markovian, i.e. the daughter is conditionally independent of the grandmother given the mother (see Materials and methods). As a result, the correlation parameter after one round of decimation is *ρ*_1_ = *p*_1_(on|on) − *p*_1_(on|off) = [*p*(on|on) − *p*(on|off)]^2^ = *ρ*^2^, where the first and last steps use the definition in Eq 2, and the middle step inserts the expressions in Eqs 5 and 6 and simplifies (see Materials and methods). Similarly, after *j* rounds of decimation we have *ρ*_*j*_ = *ρ*^*j*+1^. Because *ρ* < 1, we see that *ρ*_*j*_ → 0 as *j* → ∞. Thus, correlations vanish upon repeated rounds of decimation and renormalization. This means that correlations are not expected to change the critical properties of the distribution *P*(*n*).

The above intuition only holds if the correlations are sufficiently short-range. Indeed, Eqs 5 and 6 assume that the correlations are minimally short-range, namely Markovian. In general, it has been shown that spatial correlations only affect the critical properties of percolation if they decay as a power law, specifically *C*(*r*) ∼ *r*^−*a*^ with *a* > 3/2 in 2D [18]. As seen in Fig 5B, the correlations in the experimental data are much shorter-range than a power law. This suggests that the spatial correlations that we observe in the biofilm are not sufficiently long-range to affect the critical properties. Together with Fig 4C, we conclude that spatial correlations are not sufficient to explain the experimentally observed power law dependence of *P*(*n*) over three decades [5].

### Variability in signaling fraction

If spatial correlations cannot explain the experimentally observed power law, then what can? An important feature of the experiments that is not yet accounted for in the model is variability in the on-cell fraction *ϕ*. In particular, we previously observed that the value of *ϕ* is roughly Gaussian-distributed across 12 experiments with a mean of $\bar{\phi }=0.43$ and a standard deviation of *σ*_*ϕ*_ = 0.07 (from which the standard error of $0.07/\sqrt{12}=0.02$ comes) [5]. Furthermore, subdividing each of the 12 images into either 4 or 16 equal parts with the same aspect ratio as the original image, we find that the standard deviation of the on-cell fraction across parts (averaged over all images) is *σ*_*ϕ*_ = 0.04 (4 parts) or *σ*_*ϕ*_ = 0.05 (16 parts). Because these values are similar to *σ*_*ϕ*_ = 0.07, we conclude that the variability within biofilms is similar to that across the biofilms in our experiments.

Some variability is expected from finite size effects. Specifically, in basic percolation theory, binomial statistics dictate that the standard deviation in the fraction of on-cells would be $\sqrt{N\bar{\phi }\left(1-\bar{\phi }\right)}/N=0.006$ in a biofilm with *N* = 230 × 35 = 8,050 cells. In our mechanistic model with correlations, we find that the standard deviation is similarly small at 0.009. Because these values are much smaller than the observed value of *σ*_*ϕ*_ = 0.07, we conclude that the experimental variability is not due to finite size effects alone, and that it is necessary to explicitly incorporate variability into the model.

**Fig 6:**
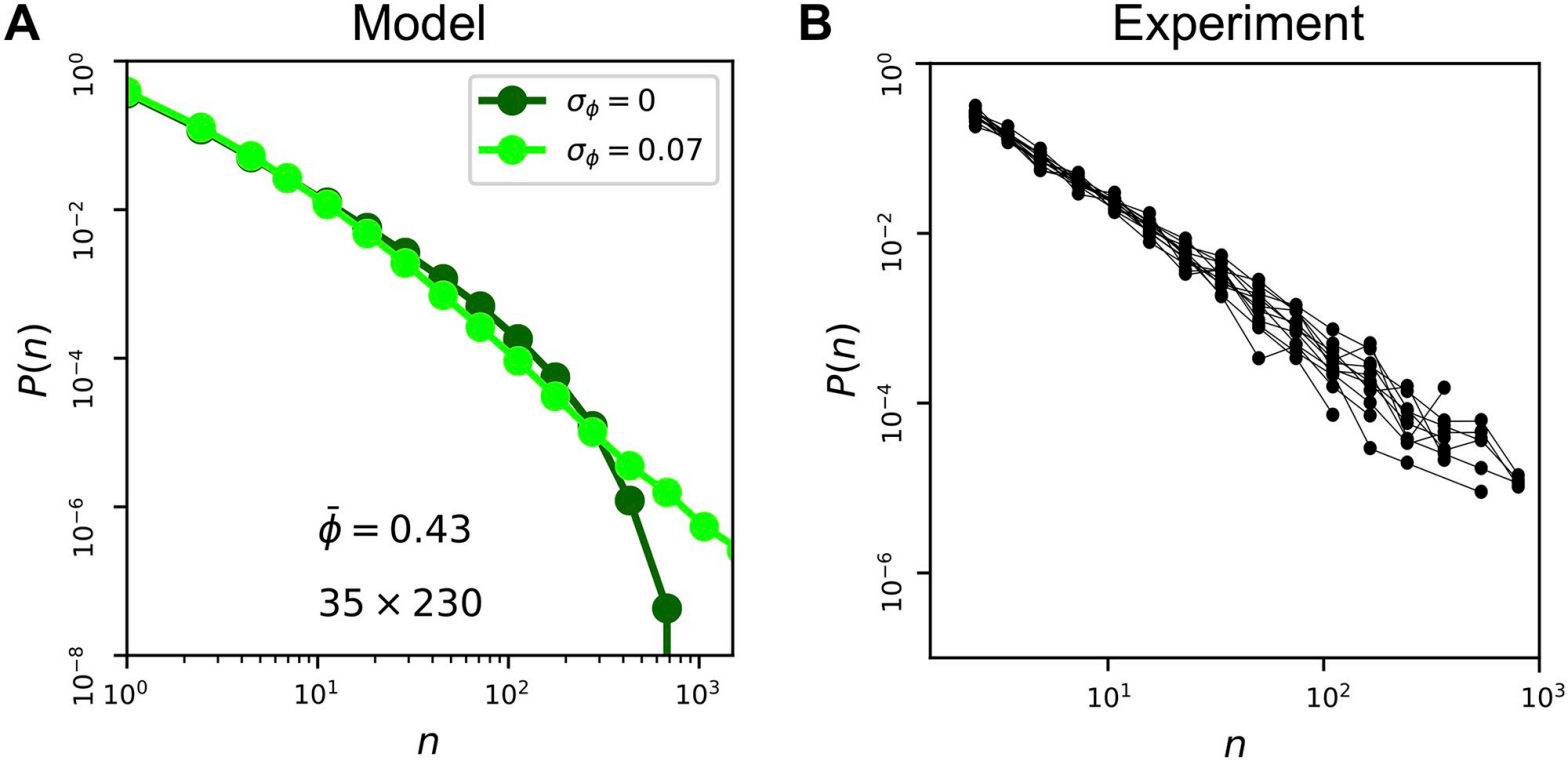
Variability can lead to power-law cluster size distribution, even for *ϕ* < *ϕ*_*c*_. (A) In the model, variability (*σ*_*ϕ*_ = 0.07) removes rolloff, causing distribution to approach a power law over three decades (*N* = 10^3^ lattices). (B) In the experiments, cluster size distributions from individual biofilms are power laws without significant rolloff, consistent with the model and the fact that we find variability in *ϕ* within each biofilm. Data are from [5] but processed individually for each biofilm.

To incorporate variability in the on-cell fraction, we draw *ϕ* for each lattice from a Gaussian distribution with standard deviation *σ*_*ϕ*_. Because we have found that correlations have little effect on *P*(*n*), we set *ρ*_div_ = 0 from here on for simplicity. The results are shown in Fig 6A, and we see that *σ*_*ϕ*_ has a significant effect on the distribution. In particular, for the experimental value *σ*_*ϕ*_ = 0.07 (light green curve), we see that the exponential rolloff at large *n* is removed, extending the range of the power law out to *n* ∼ 10^3^ as observed in the experiments [5]. The intuitive reason is that a non-negligible fraction of lattices in the ensemble have *ϕ* values that are equal to or greater than $\phi_{c}^{\text{pow}}=1/2$. Because *ϕ* is higher in these lattices, they are more likely to have large clusters. Therefore, these lattices dominate the distribution at large *n*, eliminating the rolloff. Thus, variability in *ϕ* effectively widens the range of mean $\bar{\phi }$ values at which a power law distribution is observed. We conclude that the experimental variability in *ϕ* across biofilms is sufficient to explain the experimentally observed power-law distribution.

Given that we also observe variability in *ϕ* within each biofilm, to a similar degree as across biofilms, our results suggest that the cluster size distribution from each biofilm individually should follow a power law without a significant rolloff. We test this hypothesis in Fig 6B by plotting the data from [5] separately for each biofilm. We see that indeed, the individual distributions follow a power law and do not exhibit significant rolloffs. This result suggests that the mechanism we identify above, in which variability widens the range of $\bar{\phi }$ values at which a power law distribution is observed, also applies at the individual biofilm level. It also shows that the signaling statistics are reproducible from biofilm to biofilm and thus constitute a plausible feature that could be optimized for biological function, as suggested in our previous work [5].

### Model validation using mutant strain

**Fig 7:**
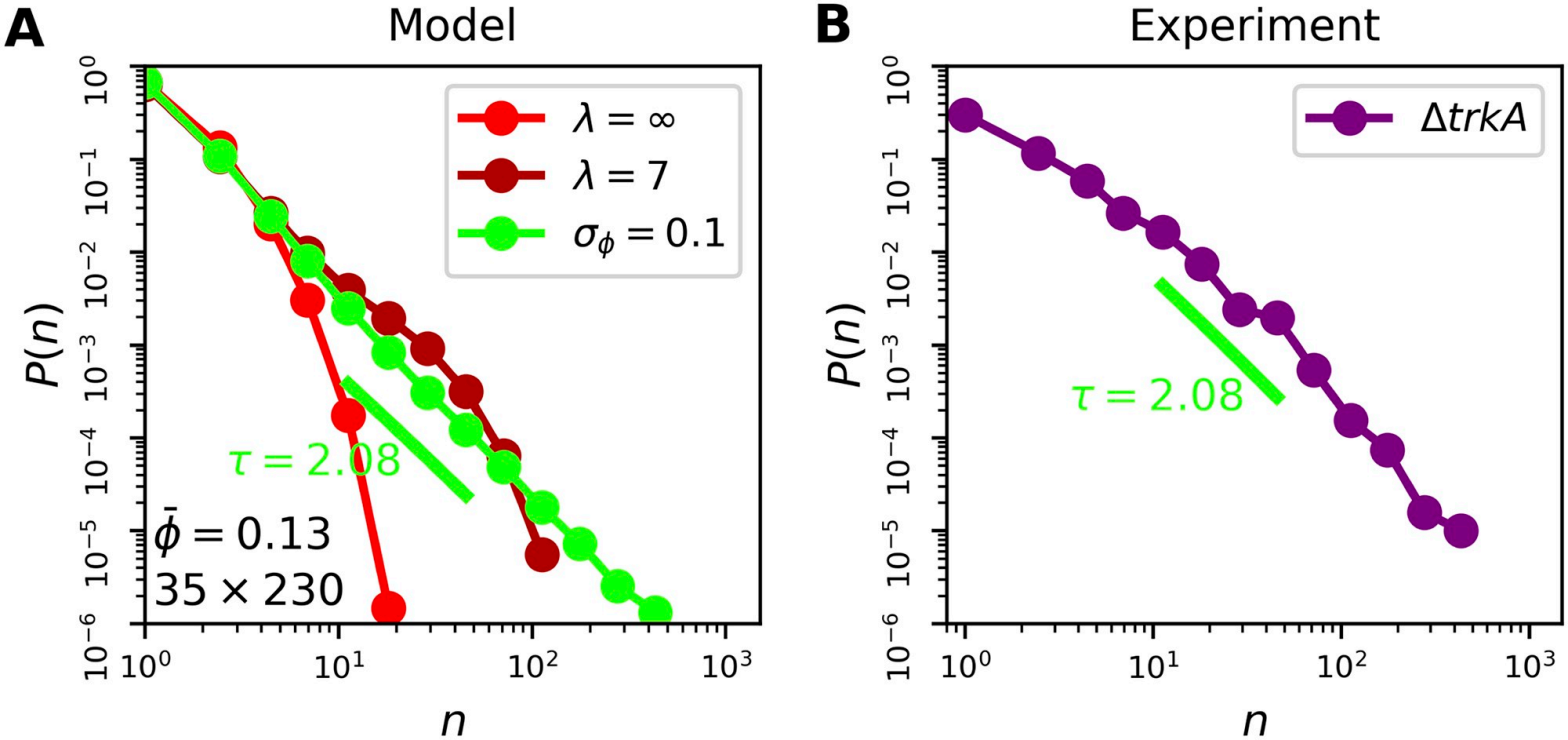
Statistics of mutant Δ*trkA* strain. (A) We progressively incorporate into the model the on-cell fraction *ϕ* = 0.13 (light red), the exponential decay of *ϕ* in space with lengthscale λ = 7 cells (dark red), and the variability *σ*_*ϕ*_ = 0.1 across lattices (green); *N* = 10^3^ lattices for each. Resulting *P*(*n*) is a power law (green) despite the fact that 0.13 is far below the critical fraction $\phi_{c}^{\text{pow}}=1/2$. (B) *P*(*n*) from Δ*trkA* data is a power law whose exponent is consistent with the model (*N* = 7 biofilms).

How can our model be tested with further experiments? One approach is to investigate a system with a different fraction of on-cells and see if our model remains valid. We previously investigated mutant strains with different on-cell fractions, including the Δ*trkA* strain with $\bar{\phi }=0.13$ and *σ*_*ϕ*_ = 0.1 [5]. As seen in Fig 7A (light red curve), basic percolation theory (*ρ*_div_ = 0, *σ*_*ϕ*_ = 0) predicts that a system with an on-cell fraction of *ϕ* = 0.13 would have a distribution of cluster sizes *P*(*n*) that is entirely exponential because 0.13 is much lower than $\phi_{c}^{\text{pow}}=1/2$.

However, the Δ*trkA* strain differs from the wild-type strain in that the fraction of on-cells is not constant in space, but rather decreases along the signaling direction [15] with a characteristic lengthscale of approximately λ = 15 *μ*m, or about 7 cell lengths [5]. The reason for this decrease is likely that the signal is dying out due to insufficient connectivity of the on-cells. Therefore, in the case of Δ*trkA*, the on-cell fraction is sufficiently low that it is likely no longer fair to treat the ability to signal and the act of signaling as equivalent. Because the experiments measure the latter, we must incorporate the observed spatial decrease into the model. To do so, we allow the on-cell fraction to vary as *ϕ*(*y*) = *ϕ*_0_*e*^−*y*/λ^, where *ϕ*_0_ is set to ensure that the spatial average of *ϕ*(*y*) is 0.13. We see in Fig 7A (dark red curve) that this feature extends the distribution to larger cluster sizes *n*. The reason is similar to that given above regarding variability: the portions of the lattice in which *ϕ* is large contain large clusters, thereby enhancing the large-*n* region of the distribution. Nonetheless, the distribution remains far from a power law in its shape. In particular, a clear exponential rolloff at large *n* is evident.

If our main finding above is correct, namely that variability in *ϕ* across biofilms is a crucial determinant of the shape of *P*(*n*), then we must also incorporate into our model the variability *σ*_*ϕ*_ = 0.1 observed for the Δ*trkA* strain. Indeed, we find that doing so has a major effect on the distribution (Fig 7A, green curve). Specifically, it removes the exponential rolloff, resulting in a power-law distribution over almost three decades. This is a strong prediction, considering that $\bar{\phi }=0.13$ is much lower than $\phi_{c}^{\text{pow}}=1/2$, and that without variability the shape is far from a power law even after accounting for the spatial dependence of *ϕ*.

To test this prediction, we measure the distribution of cluster sizes in the Δ*trkA* biofilms (see Materials and methods). Remarkably, the result, shown in Fig 7B, is a distribution that is roughly a power law over almost three decades, consistent with the model prediction. Indeed, the power-law exponent of 2.08 estimated from the model distribution via a maximum likelihood technique [19] (Fig 7A, green line) is consistent with the slope of the experimental distribution (Fig 7B, green line). This result validates our model. In particular, it supports the finding that variability of the signaling fraction across biofilms plays an important role in shaping the statistical properties of the system.

## Discussion

We have shown that experimentally observed features that go beyond the basic assumptions of percolation theory, including spatial correlations, variability, and non-uniformity, can have important consequences for signal propagation in a bacterial community. Using a mechanistic model that accounts for heritability in a cell’s propensity to participate in signaling, we have found that signal correlations decrease the fraction of participating cells needed to create a connected path, but have little effect on the cluster statistics. In contrast, variability of the signaling fraction across samples has a significant effect on the statistics, in particular producing a power-law distribution of cluster sizes at signaling fractions lower than the expected critical fraction from percolation theory. We have validated our model using a mutant strain, in particular finding that both spatial decay and variability in the signaling fraction play a crucial role in shaping the signaling statistics.

While it is clear that key observations in this system are consistent with the predictions of percolation theory (the fraction of signaling cells is very close to the percolation threshold and the cluster size distribution is a power law with the predicted exponent [5]), the deviations of other observations from either the assumptions or predictions of percolation are informative. In this sense we recognize that percolation theory is a toy model. Searching for deviations from the toy model has allowed us infer information about the biological mechanism, and then develop an enhanced model of percolation that is more appropriate. The approach of extending percolation to account for additional features has a long precedent in the literature, with variants including explosive percolation, fractional percolation, correlated percolation, bootstrap percolation, invasion percolation, and dynamical percolation [6, 20–22]. This approach is particularly suitable for biological systems, where it is natural to expect that the complexities of growth and variability may lead to observable departures from simple textbook models.

We found that incorporating the experimentally observed variability and non-uniformity of the signaling fraction into the model was necessary to explain the experimentally observed cluster statistics, whereas incorporating the experimentally observed spatial correlations in signaling was not necessary. This finding implies that certain underlying cell-level features are important in determining population-level statistical properties, whereas others are not. This categorization is consistent with approaches from statistical physics, particularly the renormalization group, which reflect the powerful notion that some microscopic details are relevant for macroscopic properties, whereas others are provably irrelevant [23]. Indeed, we explain our finding that spatial correlations do not affect the cluster statistics using a renormalization argument (Fig 5), as well as more rigorous known results from statistical physics [18]. It will be interesting to see what other cell-level features are relevant or irrelevant for capturing population-level phenomena in multicellular systems.

Finite-size effects play an important role in our results. In particular, our experimental observation window is sufficiently short in the signaling direction (∼35 cells) that spatial correlations in the signaling propensity have a measurable effect on the connectivity (Fig 4). Yet, the window is wide perpendicular to signaling (∼230 cells), and thus the window area is sufficiently large that the spatial correlations have little effect on the cluster size statistics. This choice of window size follows from experimental constraints and the desire to focus on the short and wide biofilm edge, where signaling is most important for function [15]. Nonetheless, it is an interesting open question how the finite size and aspect ratio of the system set distinct thresholds for the relevance of correlations to the connectivity and cluster statistics.

Dimensionality also plays an important role in our results. Because the biofilm edge is where cell growth is most pronounced, it is quasi-two-dimensional. In fact, biofilm formation itself can promote cell spreading via osmotic pressure gradients, reducing biofilm thickness at the edge [24]. This is part of the reason that our experiments have focused on 2D monolayers of cells. However, the properties of percolation theory depend critically on the dimensionality of the system [6]. In particular, the percolation threshold is generally smaller in 3D lattices than in 2D lattices [25] because there are more available paths for the signal to take. This observation suggests that a lower fraction of signaling cells is necessary in the bulk of the biofilm than at its edge. This prediction is currently difficult to test, as the 2D nature of our experiments is crucial for obtaining fluorescence data at the single-cell level.

The fact that spatial correlations lower the connectivity threshold in a finite system may help explain why the biofilm has an on-cell fraction of *ϕ* = 0.43 ± 0.02 [5]. Naive percolation theory predicts a threshold of $\phi_{c}^{\text{conn}}=1/2$ [6], which the biofilm does not meet. Accounting for finite-size effects lowers the threshold to $\phi_{c}^{\text{conn}}=0.45$ [5] (Fig 4), which the biofilm barely meets. Accounting for correlations lowers the threshold further to $\phi_{c}^{\text{conn}}=0.4$ (Fig 4), which the biofilm meets comfortably. Thus, correlations provide some leeway between the necessary and observed signaling fraction, which may enhance the reliability of signaling or make it robust to errors.

Although we observe spatial correlations in the signaling activity, and the results are consistent with a model that assumes inheritance of the signaling state, the inheritance mechanism is unknown. *B. subtilis* cells maintain phenotypic states through intracellular genetic networks that control the production of transcription factors [26]. Moreover, the inheritance of transcription factors and other proteins from parent cells to daughter cells can maintain specific cell types for several generations, leading to spatial correlation of cell types [27]. *B. subtilis* has even evolved the ability to control the number of generations over which certain phenotypic states are maintained [28]. Such a mechanism could drive the inheritance in signaling state that we observe here: the transcription factors regulating the expression or non-expression of ion channels, for example, could be passed from mother to daughter.

The effect of spatial correlations is a general question that is fundamental to understanding multicellular behaviors. The length scale of cell-to-cell signaling in quorum sensing bacterial communities depends on the establishment of spatial correlations [7, 29]. Moreover, the interplay of spatial heterogeneity and signaling lengthscale dictates the cooperativity of pathogenic *Pseudomonas aeruginosa* biofilms [30]. In eukaryotes, spatial correlations in cell-substrate interactions can drive collective cell migration [31], which is a fundamental multicellular process in tissue development [32] and wound healing [33].

Our study motivates further avenues of exploration in both statistical physics and cell biology. In statistical physics, our study motivates more general investigations of whether and how particular microscopic features affect macroscopic properties of percolation. The effects of spatial correlations in the site occupation probability are relatively well understood [20, 22, 34–37], whereas the effects of variability and non-uniformity in the site occupation probability are still relatively open questions [38, 39]. In cell biology, our study builds on previous work [5, 8–13] that demonstrates the utility of percolation theory as a quantitative and predictive description of multicellular phenomena. It will be interesting to see in what biological systems ideas from percolation theory will provide useful insights next.

## Materials and methods

### Experimental methods

#### Microfluidics and experimental conditions

Bacterial strains and growth conditions were as in [5]. We performed experiments in Y04D microfluidic plates using the CellASIC ONIX microfluidic system (EMD Millipore). Cells were imaged at the edge of biofilms and were confined to a single-cell layer by the PDMS structures of the microfluidic chamber. Each microscope field of view was roughly 330 *μ*m × 70*μ*m and contained 8,000 − 10,000 cells. Every 5 minutes, we took phase contrast and fluorescence images on an Olympus IX83 inverted microscope with autofocus and a 40X, 0.6 NA air objective.

To probe membrane potential, we used the cationic fluorescent dye Thioflavin-T (ThT), which acts as a Nernstian voltage indicator [15]. When cells are hyperpolarized, they retain more of the dye and have a higher signal. ThT was present in the media at a concentration of 10 *μ*M. We considered a cell to be an on-cell if its mean ThT signal exceeded a particular threshold during a signal pulse [5].

#### Computation of correlation function

To compute correlation functions, we first thresholded ThT images so that they were binary: biofilm regions above the ThT threshold would appear white and sub-threshold regions would appear black. We then applied a 2-pixel radius median filter to thresholded images so that clusters of on-cells became contiguous white regions. From this image, we created a 2D autocorrelation plot using the ImageJ command FD Math. The resulting plot was mean-subtracted and normalized such that the origin had a value of 1 and decayed to 0 away from the origin (see source code for the Radially Averaged Autocorrelation ImageJ plugin for further details).

To compute the radial autocorrelation curves (Fig 1C), we took a radial average of this 2D correlation plot. For *x* and *y* correlation curves, we took profiles of the correlation plot along the *x* and *y* axes, respectively.

To construct randomized images for such correlation computations, we took segmented biofilm images and randomly assigned a fraction of cells to be on and made them white. We then computed the autocorrelation curve on these images the same way as with the experimental images.

#### Lineage tracing for *ρ*_div_

To determine *ρ*_div_, we tracked individual cell lineages over time within biofilms using the mTrackJ imageJ plugin [40]. For each lineage, we recorded the firing state (i.e. on or off) of the parent cell and the daughter cells. Using many lineages, we computed the conditional probabilities *p*(on|on), *p*(on|off), *p*(off|on), and *p*(off|off). We then computed the order parameter *ρ*_div_ using Eq 2.

#### Spatial analysis for *ρ*_adj_

To determine *ρ*_adj_, we segmented cells in static images taken during signal pulses and determined the firing state of each cell (i.e. on or off). Because the electrical signal propagates in the direction of cell growth, cells are generally oriented along the signaling direction (Fig 1B). The adjacent cell in each case was defined as the cell whose bottom edge was closest to the given cell’s top edge, and whose centroid was within half the average cell width. We then computed the conditional probabilities *p*(on|on), *p*(on|off), *p*(off|on), and *p*(off|off) for the firing state of a cell given the state of the adjacent cell. We then computed the order parameter *ρ*_adj_ using Eq 2.

#### Image analysis for Δ*trkA*

We evaluated the cluster size distribution for Δ*trkA* biofilms in Fig 7B by first segmenting single biofilm cells in phase images using the Trainable Weka Segmentation plugin in ImageJ. We then thresholded the corresponding ThT images as described in the above section on computing correlation curves. Each contiguous white region in the thresholded image was a cluster of on-cells. We then counted how many segmented cells had the majority of their area within each cluster. The curve in Fig 7B plots the normalized histogram of these cluster sizes.

### Theoretical methods

#### Mechanistic model

To derive Eqs 3 and 4, we require that the fraction of on-cells is *ϕ* at each step in the growth process. Specifically, the rules of probability state that

$$\begin{array}{c} p\left(d\right)=\sum_{m}p\left(d,m\right)=\sum_{m}p\left(d|m\right)p\left(m\right), \end{array}$$ (7)

where *d* is the signaling state (on, off) of the daughter, and *m* is the signaling state (on, off) of the mother. Taking *d* = on and requiring that *p*(on) = *ϕ* and *p*(off) = 1 − *ϕ*, Eq 7 becomes

$$\begin{array}{c} \phi =p(\text{on}|\text{on})\phi +p(\text{on}|\text{off})(1-\phi ). \end{array}$$ (8)

Solving for *ϕ*, we obtain

$$\begin{array}{c} \phi =\frac{p(\text{on}|\text{off})}{1+p(\text{on}|\text{off})-p(\text{on}|\text{on})}. \end{array}$$ (9)

Combining this equation with Eq 2 and solving for the conditional probabilities, we obtain Eqs 3 and 4.

#### Differential growth rates

We previously observed that signal participation reduces the cell elongation rate [5], implying that on-cells grow more slowly than off-cells. Specifically, Fig 1B of [5] shows that the elongation rate is reduced by a factor of about 4 at peak signaling activity. On-cells signal for about 20 minutes (Fig 4E of [5]), whereas pulses occur every 80 minutes or so (Fig S4 of [5]). Therefore the net growth rate ratio of on-cells to off-cells is approximately *γ* = (1/4)(20/80) + (1)(60/80) ≈ 80%.

To incorporate this feature into the model, we take the mean division time to be $\bar{\tau }$ and $\bar{\tau }/\gamma$ for off-cells and on-cells, respectively. Differential growth rates change the resulting fraction of on-cells in the lattice, and therefore Eqs 3 and 4 must be modified to maintain this fraction at *ϕ*. Specifically, using the shorthand *q* ≡ *p*(on|on) and *r* ≡ *p*(on|off) and recognizing that *p*(off|on) = 1 − *q* and *p*(off|off) = 1 − *r*, the deterministic dynamics of the number of on- and off-cells are

$$\begin{array}{c} {\dot{n}}_{\text{on}}=\gamma qn_{\text{on}}+rn_{\text{off}}, \end{array}$$ (10)

$$\begin{array}{c} {\dot{n}}_{\text{off}}=\gamma \left(1-q\right)n_{\text{on}}+\left(1-r\right)n_{\text{off}}, \end{array}$$ (11)

where time is scaled by $\bar{\tau }$. At long times, the larger of the two eigenvalues of this linear dynamical system is

$$\begin{array}{c} \lambda_{+}=\frac{1}{2}[1+\gamma q-r+\sqrt{{\left(1+\gamma q-r\right)}^{2}-4\gamma \left(q-r\right)}], \end{array}$$ (12)

and the ratio of the two components of the corresponding eigenvector gives the ratio of *n*_on_ and *n*_off_. Setting the ratio of *n*_on_ and *n*_on_ + *n*_off_ to *ϕ* obtains

$$\begin{array}{c} \phi =\frac{r}{\lambda_{+}+r-\gamma q}. \end{array}$$ (13)

Note that taking *γ* = 1 makes λ_+_ = 1, and Eq 13 recovers Eq 9. Combining Eqs 12 and 13 with *ρ* = *q* − *r* (Eq 2) and solving for *q* and *r* obtains

$$\begin{array}{c} p(\text{on}|\text{on})=\phi +\frac{\rho (1-\phi )}{1-(1-\gamma )\phi }, \end{array}$$ (14)

$$\begin{array}{c} p(\text{on}|\text{off})=\phi -\frac{\gamma \phi \rho }{1-(1-\gamma )\phi }. \end{array}$$ (15)

These expressions replace Eqs 3 and 4, respectively. This derivation ignores the differential crowding effects in the simulation due to the differential growth rates, but for *ϕ* = 0.43, *ρ* = 0.38, and *γ* = 0.8 we still find that the resulting fraction of on-cells in the lattice is 0.428 ± 0.009, which includes 0.43 within error.

In this model with differential growth rates, we find that all of the predictions of Fig 3 remain unchanged: the correlation lengths in both the *x* and *y* directions are significantly larger than random (*p* < 0.001 for both), and *ρ*_adj_ = 0.19 ± 0.02, which actually now agrees with the measured 0.17 within error.

#### Renormalization argument

To derive Eqs 5 and 6, we recognize that the conditional probability of the daughter given the mother after one round of decimation is the conditional probability of daughter given the grandmother before the decimation. Again using the rules of probability, we write the latter as

$$\begin{array}{c} p\left(d|g\right)=\sum_{m}p\left(d,m|g\right)=\sum_{m}p\left(d|m,g\right)p\left(m|g\right), \end{array}$$ (16)

where *g* is the signaling state (on, off) of the grandmother. The spatial Markovian assumption states that *d* is conditionally independent of *g* given *m*. Therefore we have *p*(*d*|*m*, *g*) = *p*(*d*|*m*), and Eq 16 becomes

$$\begin{array}{c} p\left(d|g\right)=\sum_{m}p\left(d|m\right)p\left(m|g\right). \end{array}$$ (17)

Setting *d* = on and *g* = on gives Eq 5. Setting *d* = on and *g* = off gives Eq 6.

To derive the relation *ρ*_1_ = *ρ*^2^ below Eq 6, we insert Eqs 5 and 6 into the definition *ρ*_1_ = *p*_1_(on|on) − *p*(on|off). Again using the shorthand *q* ≡ *p*(on|on) and *r* ≡ *p*(on|off) and recognizing that *p*(off|off) = 1 − *q* and *p*(off|off) = 1 − *r*, this insertion obtains

$$\begin{array}{c} \rho_{1}=q^{2}+r\left(1-q\right)-qr-r\left(1-r\right)=q^{2}-2qr+r^{2}={\left(q-r\right)}^{2}. \end{array}$$ (18)

Because *ρ* = *q* − *r* (Eq 2), we see that *ρ*_1_ = *ρ*^2^.
